# Incorporating competing risk theory into evaluations of changes in cancer survival: making the most of cause of death and routinely linked sociodemographic data

**DOI:** 10.1186/s12889-020-09084-8

**Published:** 2020-06-26

**Authors:** Cameron M. Wright, Anna K. Nowak, Georgia Halkett, Rachael E. Moorin

**Affiliations:** 1 Health Economics and Data Analytics, Faculty of Health Sciences, School of Public Health, Curtin University, Kent St, Bentley, 6102 Western Australia; 2 School of Medicine, College of Health & Medicine, University of Tasmania, Churchill Avenue, Hobart, Tasmania 7005 Australia; 3 Department of Medical Oncology, Sir Charles Gairdner Hospital, Hospital Ave, Nedlands, 6009 Western Australia; 4 School of Nursing, Midwifery and Paramedicine, Faculty of Health Sciences, Curtin University, Kent St, Bentley, 6102 Western Australia; 5 Midwifery and Paramedicine, Faculty of Health Sciences, School of Nursing, Curtin University, Kent St, Bentley, 6102 Western Australia; 6 Centre for Health Services Research, Faculty of Medicine, Dentistry and Health Sciences, School of Population and Global Health, University of Western Australia, 35 Stirling Highway, Crawley, 6009 Western Australia

**Keywords:** Cancer survival, Competing risks, Relative survival

## Abstract

**Background:**

Relative survival is the most common method used for measuring survival from population-based registries. However, the relative survival concept of ‘survival as far as the cancer is concerned’ can be biased due to differing non-cancer risk of death in the population with cancer (competing risks). Furthermore, while relative survival can be stratified or standardised, for example by sex or age, adjustment for a broad range of sociodemographic variables potentially influencing survival is not possible. In this paper we propose Fine and Gray competing risks multivariable regression as a method that can assess the probability of death from cancer, incorporating competing risks and adjusting for sociodemographic confounders.

**Methods:**

We used whole of population, person-level routinely linked Western Australian cancer registry and mortality data for individuals diagnosed from 1983 to 2011 for major cancer types combined, female breast, colorectal, prostate, lung and pancreatic cancers, and grade IV glioma. The probability of death from the index cancer (cancer death) was evaluated using Fine and Gray competing risks regression, adjusting for age, sex, Indigenous status, socio-economic status, accessibility to services, time sub-period and (for all cancers combined) cancer type.

**Results:**

When comparing diagnoses in 2008–2011 to 1983–1987, we observed substantial decreases in the rate of cancer death for major cancer types combined (*N* = 192,641, − 31%), female breast (− 37%), prostate (− 76%) and colorectal cancers (− 37%). In contrast, improvements in pancreatic (− 15%) and lung cancers (− 9%), and grade IV glioma (− 24%) were less and the cumulative probability of cancer death for these cancer types remained high.

**Conclusion:**

Considering the justifiable expectation for confounder adjustment in observational epidemiological studies, standard methods for tracking population-level changes in cancer survival are simplistic. This study demonstrates how competing risks and sociodemographic covariates can be incorporated using readily available software. While cancer has been focused on here, this technique has potential utility in survival analysis for other disease states.

## Background

Public funding of prevention, screening, treatment programs and medical research for cancer is often justified by citing improved population health. While clinical trial data are important for showing efficacy for a selected population in a trial setting, only population-based data allows the overall and incremental impact of these initiatives on the burden of cancer to be evaluated.

Survival statistics are popular for tracking the progress of cancer initiatives and spending on cancer because they appear easy to interpret (i.e. increased survival is a measure of success) [1]. However, other measures need to be considered. For example, the influence of changing incidence can be factored in by assessing age-standardised incidence-based mortality [2]. While incidence has changed with time, so has the mortality rate from non-cancer causes, these being ‘competing risks’ to cancer as a cause of death. Sociodemographic factors influencing death – both from cancer and from competing risks – also vary over time, and this change should be accounted for if attempting to isolate changes in the probability of death from cancer due to cancer control strategies.

Relative survival is the most common method used for measuring survival from population-based cancer registries. This metric is a ratio of the observed survival rate of the patients and an expected survival rate in the general population. Relative survival therefore gives an estimate of mortality in the cancer population over and above what is observed for the general population, termed excess mortality. Relative survival can introduce bias if the non-cancer mortality risk differs among the group with cancer relative to the general population (e.g. if markedly different consumption of alcohol to the general population), or as the proportion of people with cancer in the general population increases (e.g. for older persons) [3, 4]. These biases may be less important for cancer types with, on average, short follow-up time in registry data (e.g. lung cancer) [5]. While traditional survival analysis allows stratification of measures, for example by age group or sex, stratification or adjustment for sociodemographic covariates is difficult, requiring the availability or construction of specialised life tables (e.g. used in [6]).

Fine and Gray multivariable regression models [7] provide a means to consider the probability of death from cancer over time, allowing for incorporation of competing risks and adjustment for sociodemographic variables [8]. The aim of this study was to evaluate the utility of Fine and Gray competing risks regression to analyse the probability of death from cancer in the context of changing cancer incidence in Western Australia (WA) from 1983 to 2011.

## Methods

The reporting of this population-based retrospective cohort study is based on the REporting of studies Conducted using Observational Routinely-collected health Data (RECORD) statement [9].

### Data sources and linkage

Person-level routinely linked data for individuals diagnosed with cancer in WA between 1 January 1983 and 31 December 2011 were extracted from the WA Cancer Registry, WA Death Registrations and the WA Hospital Morbidity Data Collection, via the WA Data Linkage System [10]. These data were used in a previous study evaluating changes in prevalence of cancer [11].

### Description of participants

Incident cancers were included on the basis of tumour site code, morphology code and behaviour type. WA residents with a diagnosis of any invasive primary cancer (excluding metastases from a previous primary cancer, benign and in situ neoplasms) were included. Individual cancer types were classified using the International Classification of Diseases for Oncology code [12], or morphology / tissue type code (for grade IV glioma) provided in the cancer registry data (Additional File 1). The earliest record (index cancer) was included if the same primary cancer type was recorded more than once for the same individual.

A mix of high- to low-incidence cancers with variable survival profiles were selected: major cancer types combined, and separately: female breast, colorectal, lung, prostate and pancreatic cancers, and grade IV glioma.

The cancer types constituting major cancer types combined in this analysis constitute the bulk of cancer types reported through the registry. Different cancer types recorded for the same individual were included for the major cancer types combined analysis and considered as separate overlapping events with the incident record identified for each cancer type.

### Outcomes, exposure and covariates

The available years of diagnosis were divided equally into six sub-periods: 1983–87; 1988–92; 1993–97; 1998–2002; 2003–07; 2008–2011.

Cause of death was divided into ‘primary cancer’ (hereafter referred to as cancer death) or ‘other’ (which included non-cancer causes, or primary cancers in a different site) using a dedicated cancer registry variable. Follow up was from the date of diagnosis to the first of recorded date of death or 18 November 2012, inclusive.

Age at diagnosis, sex, Indigenous status, census-specific postcode-based Socio-economic Index for Areas (SEIFA) quintiles of relative socioeconomic disadvantage [13], and access to health services using the Accessibility and Remoteness Index of Australia [14] were also extracted. Comorbidity was ascertained from the hospital record most closely aligned with the date of the incident cancer record using the Multipurpose Australian Comorbidity Scoring System (MACSS, incorporating 102 comorbid conditions) [15]. Comorbidity was specified as the number of the conditions specified in MACSS, excluding cancer, as a continuous variable. Hospital data are available in the WA Data Linkage System for all separations from 1970; however, since the data used here were part of a larger study this was limited to the analysis of hospital use occurring on or after 1 January 1998 [11].

### Statistical analysis

Patient characteristics at diagnosis were compared using the Kruskal–Wallis test for continuous variables and the Chi-squared test for categorical variables. Sex-specific age-standardised incidence rates for each time period were calculated using WA population estimates from the first sub-period as a reference [16]. Age- and sex-adjusted Poisson regression was used to assess differences relative to the first time period.

Fine and Gray competing risks regression [7] was performed to estimate the cumulative incidence of cancer death, for major cancer types combined and for specific cancer types separately using death from the index cancer as the primary failure event and death from any other cause, including subsequent cancer diagnoses, as the competing risk. Since hospital data were only available from 1 January 1998, modelling for cancer death with and without comorbidity was undertaken for the following sub-time periods 1998–2002; 2003–2007; 2008–2011. All models were adjusted for age at diagnosis, sex, Indigenous status, socio-economic status, accessibility to services and calendar time period of incident cancer diagnosis. Cancer type was also adjusted for in the model for major cancer types combined.

Competing risks analysis accounts for the potential imbalance observed in standard Cox regression when subjects who are lost to follow up and those who have died of non-cancer causes (or not the cancer of interest) are considered equivalent from a statistical perspective. Fine and Gray proposed an alternative model that keep subjects who experience competing events ‘at risk’ in the model so that they can be counted as not having any chance of failing, rather than treating these subjects as censored [7]. Austin and Fine have published on the practical application of Fine and Gray competing risks regression, including interpretation of model outputs [17]. Where competing risks are present, the use of standard Cox regression may over-estimate the incidence of the outcome of interest [17]. Fine and Gray competing risks regression allows direct estimation of the effect of model covariates on the cumulative incidence function (i.e. probability of the outcome of interest over time) making this more appropriate for prediction [18]. The model was modified to enable robust standard errors to account for correlation within multiple records of the same person using clustering on the unique person identifier. The assumption of proportionality of sub-distribution hazards for the Fine and Gray model was tested by evaluating the log (−log) transformation of the non-parametric cumulative incidence function estimators stratified by exposure variable (i.e. time sub-period).

The sub-distribution hazard ratios (SHRs) for model covariates indicate the relative rate of cancer death among those still alive or who have experienced a competing event [17]. The models were then used to determine changes in the probability of cancer death (using the cumulative incidence function) at various times after diagnosis (using actual follow up and limited, modelled out-of-sample extrapolation, where necessary) for those diagnosed with: (i) all major cancer types combined, and; (ii) specific cancers for each according to calendar time period of diagnosis holding other covariates at their mean. The cumulative probability of cancer death is directly related to the SHRs and is more intuitive than the SHRs to interpret.

All *p*-values are two sided. Statistical analyses were performed using Stata SE (Version 14.1, College Station, Texas).

## Results

Over the study period 192,641 individual cancer diagnoses were included; 88% of the total 218,203 for all cancers reported in WA (Table 1, Additional File 2). Age was missing for 5 (0.00%) and Indigenous status missing for 89 (0.05%) of total diagnoses; these cases were dropped from the Fine and Gray models. The cohort were more likely to be male (55%), aged 65–84 years (48%), and live in an area highly accessible to healthcare services (85%). Exceptions to this trend were for female breast cancer where the highest proportion of diagnoses were among women aged 45–64 years and grade IV glioma, where those aged 45–64 and 65–84 years at diagnosis each accounted for 44% of cases. Lung cancer diagnosed from 2008 to 2011 had approximately equal proportions in the higher and lower socioeconomic groups, representing a shift from earlier time periods where the more disadvantaged groups were proportionately higher.

**Table 1:** Characteristics of the cohort

Characteristic		Time period of diagnosis												***p*** value*
		1983–1987		1988–1992		1993–1997		1998–2002		2003–2007		2008–2011		
		n	%	n	%	n	%	n	%	n	%	n	%	
**Major cancer types combined**														
Follow up in years	Median (IQR)	2.50 (13.78)		3.58 (17.47)		6.01 (14.47)		6.94 (9.92)		4.79 (5.38)		1.36 (2.01)		< 0.001
Number of comorbidities at Dx	Mean (SD)							1.87 (1.82)		1.70 (1.85)		1.51 (1.73)		< 0.001
Sex	Female	9383	47.2	11,730	47.9	13,804	44.1	16,155	46.1	18,528	43.8	16,959	42.8	< 0.001
Age group at Dx	0-10 yrs	113	0.6	117	0.5	128	0.4	145	0.4	129	0.3	131	0.3	
	11-17 yrs	77	0.4	72	0.3	123	0.4	108	0.3	118	0.3	90	0.2	
	18-24 yrs	246	1.2	239	1.0	272	0.9	277	0.8	313	0.7	262	0.7	
	25-44 yrs	2132	10.7	2746	11.2	3074	9.8	3341	9.5	3441	8.1	3006	7.6	
	45-64 yrs	6635	33.4	7859	32.1	10,151	32.4	11,983	34.2	15,297	36.2	14,856	37.5	
	65-84 yrs	9676	48.7	11,920	48.7	15,553	49.7	16,596	47.4	19,926	47.1	18,230	46.0	
	85+ yrs	998	5.0	1524	6.2	1984	6.3	2585	7.4	3081	7.3	3082	7.8	< 0.001
**Total**		**19,882**	**10.3**	**24,477**	**12.7**	**31,285**	**16.2**	**35,035**	**18.2**	**42,305**	**22.0**	**39,657**	**20.6**	
**Female breast cancer**														
Follow up in years	Median (IQR)	8.81 (21.05)		14.01 (16.11)		14.43 (10.55)		10.72 (4.12)		5.93 (2.81)		1.77 (1.97)		< 0.001
Number of comorbidities at Dx	Mean (SD)							1.14 (1.43)		0.95 (1.37)		0.85 (1.28)		< 0.001
Age group at Dx	25-44 yrs	463	17.8	634	18.4	710	16.2	737	14.1	776	13.3	694	12.6	
	45-64 yrs	1071	41.2	1497	43.5	2025	46.2	2689	51.3	3110	53.4	2821	51.0	
	65-84 yrs	939	36.1	1129	32.8	1441	32.9	1567	29.9	1662	28.6	1720	31.1	
	85+ yrs	122	4.7	179	5.2	197	4.5	237	4.5	270	4.6	287	5.2	< 0.001
**Total**		**2599**	**9.6**	**3444**	**12.8**	**4379**	**16.2**	**5238**	**19.4**	**5820**	**21.5**	**5527**	**20.5**	
**Colorectal cancer**														
Follow up in years	Median (IQR)	2.56 (12.68)		3.50 (13.83)		4.16 (13.48)		5.69 (9.45)		4.62 (4.99)		1.42 (1.98)		< 0.001
Number of comorbidities at Dx	Mean (SD)							2.24 (1.97)		2.16 (1.95)		2.11 (1.85)		0.49
Sex	Female	1476	47.8	1711	47.7	1910	44.9	2202	44.2	2418	44.6	2193	42.9	< 0.001
Age group at Dx	25-44 yrs	130	4.2	171	4.8	182	4.3	213	4.3	207	3.8	223	4.4	
	45-64 yrs	1068	34.6	1172	32.7	1373	32.3	1528	30.7	1751	32.3	1680	32.9	
	65-84 yrs	1692	54.8	1969	54.9	2322	54.6	2719	54.6	2910	53.7	2670	52.3	
	85+ yrs	193	6.3	271	7.6	369	8.7	510	10.2	534	9.8	522	10.2	< 0.001
**Total**		**3088**	**11.7**	**3586**	**13.6**	**4254**	**16.1**	**4978**	**18.8**	**5423**	**20.5**	**5110**	**19.3**	
**Lung cancer**														
Follow up in years	Median (IQR)	0.44 (1.09)		0.53 (1.27)		0.53 (1.29)		0.59 (1.34)		0.64 (1.65)		0.55 (1.10)		< 0.001
Number of comorbidities at Dx	Mean (SD)							2.16 (1.85)		2.01 (1.87)		1.87 (1.79)		< 0.001
Sex	Females	711	25.0	911	29.4	1056	31.5	1326	34.8	1632	37.8	1566	40.6	< 0.001
Age group at Dx	25-44 yrs	59	2.1	70	2.3	65	1.9	79	2.1	63	1.5	65	1.7	
	45-64 yrs	1096	38.6	1036	33.4	951	28.3	1027	26.9	1146	26.5	1073	27.8	
	65-84 yrs	1596	56.2	1857	59.9	2137	63.7	2431	63.7	2753	63.8	2292	59.4	
	85+ yrs	89	3.1	136	4.4	198	5.9	272	7.1	348	8.1	418	10.8	< 0.001
**Total**		**2841**	**13.3**	**3102**	**14.6**	**3357**	**15.8**	**3814**	**17.9**	**4317**	**20.3**	**3857**	**18.1**	
**Prostate Cancer**														
Follow up in years	Mean (SD)	5.55 (6.11)		6.98 (6.50)		9.89 (6.04)		8.04 (4.07)		5.44 (2.14)		1.90 (1.16)		< 0.001
Number of comorbidities at Dx	Mean (SD)							1.92 (1.72)		1.35 (1.68)		1.04 (1.42)		< 0.001
Age group at Dx	25-44 yrs	5	0.3	< 5	0.2	6	0.1	14	0.3	36	0.5	49	0.6	
	45-64 yrs	248	14.1	402	15.5	1408	26.0	1526	32.6	2875	37.4	3258	41.1	
	65-84 yrs	1364	77.3	1911	73.8	3680	67.9	2822	60.2	4378	56.9	4258	53.8	
	85+ yrs	147	8.3	272	10.5	323	6.0	322	6.9	408	5.3	352	4.4	< 0.001
**Total**		**1765**	**5.9**	**2589**	**8.6**	**5417**	**18.0**	**4684**	**15.6**	**7697**	**25.6**	**7919**	**26.3**	
**Pancreatic cancer**														
Follow up in years	Median (IQR)	0.18 (0.48)		0.23 (0.49)		0.31 (0.63)		0.35 (0.73)		0.38 (0.83)		0.44 (0.91)		< 0.001
Number of comorbidities at Dx	Mean (SD)							2.33 (1.94)		2.41 (2.12)		2.03 (1.89)		
Sex	Females	218	46.0	261	49.3	331	47.8	413	51.1	504	49.0	448	46.7	0.42
Age group at Dx	25-44 yrs	15	3.2	15	2.8	23	3.3	28	3.5	28	2.7	27	2.8	
	45-64 yrs	124	26.2	138	26.1	167	24.1	215	26.6	306	29.7	268	27.9	
	65-84 yrs	313	66.0	321	60.7	425	61.4	461	57.1	566	55.0	530	55.3	
	85+ yrs	22	4.6	53	10.0	76	11.0	101	12.5	129	12.5	134	14.0	< 0.001
**Total**		**474**	**10.6**	**529**	**11.8**	**692**	**15.4**	**808**	**18.0**	**1029**	**22.9**	**959**	**21.4**	
**Grade IV glioma**														
Follow up in years	Median (IQR)	0.42 (0.89)		0.48 (0.81)		0.42 (0.79)		0.52 (1.49)		0.59 (1.08)		0.64 (0.84)		0.0170
Number of comorbidities at Dx	Mean (SD)							2.39 (1.81)		2.66 (2.02)		2.75 (1.95)		0.14
Sex	Females	71	45.8	99	44.8	107	42.8	119	38.3	126	38.2	118	43.7	0.34
Age group at Dx	25-44 yrs	20	12.9	20	9.0	20	8.0	28	9.0	16	4.8	12	4.4	
	45-64 yrs	71	45.8	109	49.3	104	41.6	131	42.1	146	44.2	118	43.7	
	65-84 yrs	61	39.4	81	36.7	110	44.0	137	44.1	154	46.7	130	48.1	
	85+ yrs	0	0.0	< 5	1.4	10	4.0	11	3.5	10	3.0	7	2.6	0.018
**Total**		**155**	**10.1**	**221**	**14.4**	**250**	**16.3**	**311**	**20.2**	**330**	**21.5**	**270**	**17.6**	

*Dx* Diagnosis, *SD* Standard deviation, *IQR* Interquartile range, *n* number of cases, *%* percentage of total cancer type as specified

*Two-sided *p* values calculated using Chi squared for categorical variables and Kruskal-Wallis test for continuous variables. This assessed changes between variables over the time period

Cell counts with less than 5 cases are shown as < 5 to preserve anonymity

Note the number of individuals aged under 25 years of age is not shown for the individual cancer types due to very low numbers

The age-standardised incidence rate of major cancer types combined increased by 9.5% overall over the study period with greater increases observed in males (+ 14.2%) compared with females (+ 4.5%) (Table 2). For individual cancers, the largest increases in age standardised incidence were observed for prostate cancer (+ 153.5%), lung cancer in females (+ 34%) and female breast cancer (+ 31%). Lung cancer showed the largest disparity across sexes being the cancer having the largest increase in females (+ 34.3%) and largest reduction in males (− 40.7%). Disparate changes in the incidence of colorectal cancer were also observed, with a modest reduction observed in females (− 10.8%, *p* < 0.001) but no change in males (+ 2.9%, *p* = 0.534). Similarly, pancreatic cancer showed a large increase in incidence in females (+ 21%, *p* = 0.015).

**Table 2:** Changes in the age (and sex where relevant) standardised incidence rate of cancer in Western Australia according to cancer type and sex

Sex	Type of Cancer	Time period of diagnosis											Change over study period*	Change over study period (1983–1987 to 2008–2011)	
		1983–1987	1988–1992		1993–1997		1998–2002		2003–2007		2008–2011				
		rate per 100,000	rate per 100,000	% of prev rate	rate per 100,000	% of prev rate	rate per 100,000	% of prev rate	rate per 100,000	% of prev rate	rate per 100,000	% of prev rate	rate per 100,000	% of rate in first time period	***p*** value^**#**^
**Females**	Major cancer types combined	260.6	273.4	4.9	274.9	0.5	274.2	−0.3	273.2	−0.4	272.2	−0.4	11.6	4.5	0.001
	Female breast cancer	73.6	83.3	13.2	91.9	10.3	94.4	2.7	92.3	−2.2	96.6	4.7	23.0	31.3	< 0.001
	Colorectal cancer	41.8	41.0	−1.9	39.4	−3.9	39.1	−0.8	37.9	−3.1	37.3	−1.6	−4.5	−10.8	0.001
	Lung cancer	20.1	22.0	9.5	22.0	0.0	23.9	8.6	25.8	7.9	27.0	4.7	6.9	34.3	< 0.001
	Pancreatic cancer	6.2	6.2	0.0	6.8	9.7	7.3	7.4	7.7	5.5	7.5	−2.6	1.3	21.0	0.015
	Glioma grade IV	2.0	2.4	20.0	2.2	−8.3	2.2	0.0	2.0	−9.1	2.1	5.0	0.1	5.0	0.84
**Males**	Major cancer types combined	282.5	282.0	−0.2	322.8	14.5	293.3	−9.1	313.3	6.8	322.6	3.0	40.1	14.2	< 0.001
	Colorectal cancer	44.8	43.8	−2.2	46.3	5.7	46.8	1.1	43.5	−7.1	46.1	6.0	1.3	2.9	0.534
	Lung cancer	59.2	51.2	−13.5	45.4	−11.3	42.1	−7.3	38.5	−8.6	35.1	−8.8	−24.1	−40.7	< 0.001
	Prostate cancer	49.0	59.4	21.2	106.5	79.3	78.4	−26.4	110.4	40.8	124.2	12.5	75.2	153.5	< 0.001
	Pancreatic cancer	7.1	6.2	−12.7	7.1	14.5	6.7	−5.6	7.6	13.4	7.9	3.9	0.8	11.3	0.15
	Glioma grade IV	2.3	2.9	26.1	2.9	0.0	3.3	13.8	3.0	−9.1	2.5	−16.7	0.2	8.7	0.59
**Persons**	Major cancer types combined	271.7	277.7	2.2	299.1	7.7	283.8	−5.1	293.4	3.4	297.6	1.4	25.9	9.5	< 0.001
	Female breast cancer	73.6	83.3	13.2	91.9	10.3	94.4	2.7	92.3	−2.2	96.6	4.7	23.0	31.3	< 0.001
	Colorectal cancer	43.3	42.4	− 2.1	42.9	1.2	43.0	0.2	40.7	−5.3	41.7	2.5	−1.6	−3.7	0.073
	Lung cancer	39.8	36.7	−7.8	33.8	−7.9	33.1	−2.1	32.2	−2.7	31.1	−3.4	−8.7	−21.9	< 0.001
	Prostate cancer	49.0	59.4	21.2	106.5	79.3	78.4	−26.4	110.4	40.8	124.2	12.5	75.2	153.5	< 0.001
	Pancreatic cancer	6.6	6.2	−6.1	6.9	11.3	7.0	1.4	7.6	8.6	7.7	1.3	1.1	16.7	0.007
	Glioma grade IV	2.2	2.7	22.7	2.6	−3.7	2.8	7.7	2.5	−10.7	2.3	−8.0	0.1	4.5	0.617

% of previous rate = difference in incidence rate of cancer as a percentage of the rate in the previous time period [((Rate in period 2 - Rate in period 1)/Rate in period 1)*100]

*Change in rate over study period = Rate in 2008–2011 - Rate in 1983–1987 time period

% change over study period = Difference in incidence rate in final time period as a percentage of the rate in the first time period

^#^*p* value of difference in incidence rate over study period evaluated using Poisson regression adjusted for age and sex where relevant

Negative values denote a reduction in the incidence rate

A decreased rate of cancer death for major cancer types combined of 31% (*p* < 0.001) was observed, when comparing the most recent sub-period (2008–2011) to the earlier sub-period (Table 3). While there were also observed relative decreases for all individual cancer types, this reduction was markedly less for lung (9%, *p* < 0.001) and pancreatic cancers (15%, *p* = 0.035), and grade IV glioma (24%, *p* = 0.024). For individual cancers, the largest decreases were observed for prostate cancer death (76%, *p* < 0.001) and female breast cancer death (63%, *p* < 0.001). A 37% decrease in the rate of colorectal cancer death was also observed (*p* < 0.001).

**Table 3:**
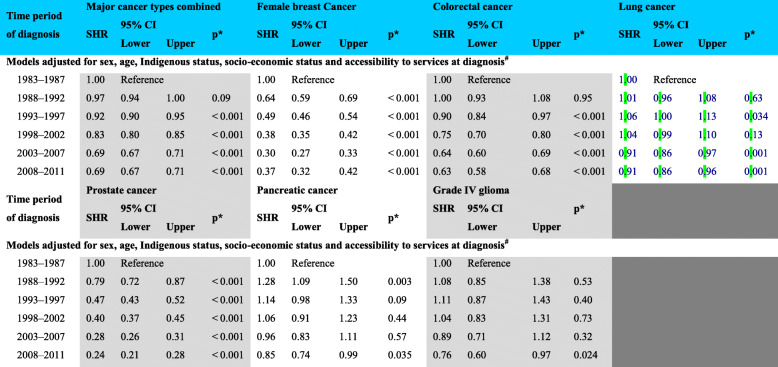
Competing risk regression analysis of Western Australia cancer-specific mortality by time period of diagnosis for major cancer types combined and six selected cancer types

SHR = sub-distribution hazard ratio of cancer death (of specific cancer types for models with one cancer type)

*Fine and Gray competing risks regression model. All *p*-values are two-sided. #Analysis for all incident cancers was also adjusted for cancer type

Because comorbidity could only be adjusted for in the most recent three sub-periods, SHRs for models with and without comorbidity as a covariate have been provided in Additional File 3. In comparing to the baseline sub-period (1998–2002), adding comorbidity moved the negative SHRs towards the null for the 2008–2011 sub-period for major cancer types combined, lung, prostate and pancreatic cancers, with only a minor increase for colorectal (0.86 to 0.88) and a decrease in the SHR for grade IV glioma (0.73 to 0.69). For the 2003–2007 sub-period, the SHR for prostate cancer increased from 0.74 to 0.79 with the addition of comorbidity. The addition of comorbidity changed the SHR for grade IV glioma from 0.86 (95% CI 0.72 to 1.02) to 0.77 (95% CI 0.65 to 0.92) for the 2003–2007 sub-period. There was little change to other SHRs for the 2003–2007 sub-period.

**Fig. 1:**
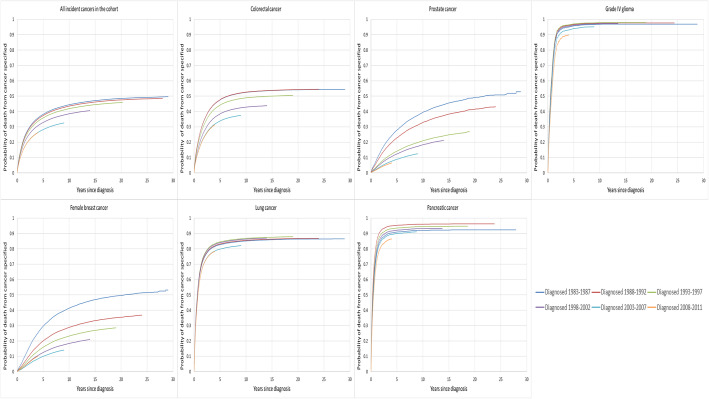
Adjusted* cumulative probability of death from index cancer^#^ in Western Australia for major cancer types combined and selected cancers diagnosed 1983 to 2011, by sub-period. *Age, sex, period, Indigenous status, socioeconomic quintile, accessibility to health services and, for the major cancer types combined analysis, cancer type. Covariates are held at the mean of the observations in the respective cancer cohort used in the model. Thus the probability of cancer death is adjusted for these factors across each time period. Note curves show within sample estimations (i.e. no extrapolation of the probability of death beyond the follow-up time is shown in this figure). ^#^Index cancer refers to the first invasive primary cancer of each type

Figure 1 shows the adjusted probability of cancer death against time following a primary cancer diagnosis, in the presence of competing risks from other causes. For the majority of cancers the largest change in the probability of cancer death (i.e. slope of the cumulative incidence function curve) between time periods occurred in the first year following diagnosis. For lung and pancreatic cancers and grade IV glioma the between-sub-period changes are less overall, with steeper increases in the cumulative probability of cancer death with time compared to other cancer types.

**Fig. 2:**
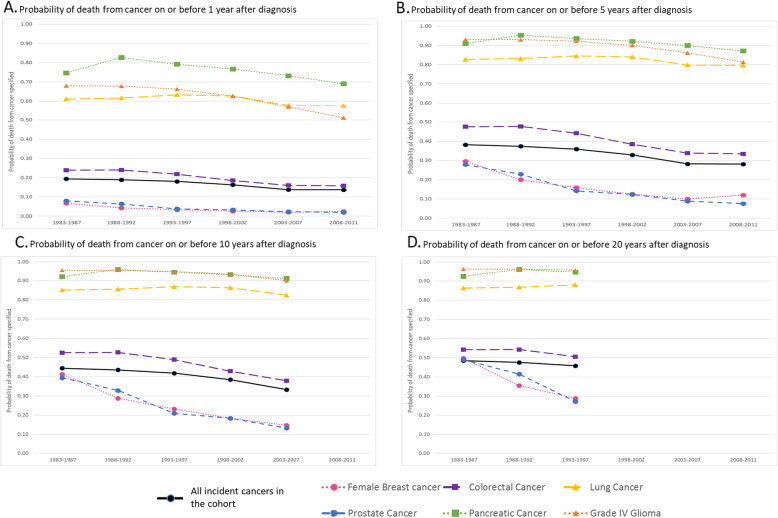
Adjusted* cumulative probability of death from index cancer^#^ in Western Australia for major cancer types combined and selected cancers diagnosed 1983 to 2011, by cancer type. *Age, sex, period, Indigenous status, socioeconomic quintile, accessibility to health services and, for the major cancer types combined analysis, cancer type. Covariates are held at the mean of the observations in the respective cancer cohort used in the Fine and Gray model. Thus the probability of death is adjusted for these factors across each time period. The figure shows both the within-sample and out-of-sample (i.e. beyond the post-diagnosis follow-up time of the data) estimations of 1 year for each of the following: 2008–2011 sub-period for panel B, 2003–2007 sub-period for panel C, 1993–1998 sub-period for panel D. ^#^Index cancer refers to the first invasive primary cancer of each type

Figure 2 compares the probabilities of cancer death at 1, 5, 10 and 20 years following diagnosis by cancer types across sub-periods. One to four years out-of-sample extrapolation is included in panel b, c and d to allow 5, 10 and 20 year probabilities to be reported for the respective time periods of 2008–2011, 2003–2007 and 1993–1997. The probability of cancer death was lowest for female breast and prostate cancer, relative to other cancer types, in stark contrast to pancreatic and lung cancers, and grade IV glioma where the cumulative probability of death was uniformly high. For all cancer types except for lung cancer, there were reductions in the probability of death at or before 1 year post-diagnosis. There was little change beyond 5 years post-diagnosis for any cancer type.

## Discussion

Unlike similar studies from different settings [19, 20], we have analysed changes in cancer incidence alongside changes in the probability of cancer death. This is in the context of competing risks of death from other causes, and adjusting for sociodemographic confounders.

With reference to relative survival, Talback and Dickman [3] have demonstrated that biases introduced by differential mortality risk profiles among people with cancer can be large for older age groups or common cancers, and when combined can produce substantial error. While the major advantage of a relative survival approaches is that cause of death information is not required, where this information is available incorporating competing risks provides a conceptually improved way of analysing temporal changes. While this study did not aim to prove quantitative superiority of competing risks regression, an assessment of the face validity of changes observed is helpful to correlate observed trends with known changes to cancer prevention, detection and management.

The largest observed increases in age-standardised incidence and reduction in the probability of cancer death were seen in prostate cancer, with the greatest change in seen between those diagnosed in 1988–1992 and those diagnosed in 1993–1997. There was increased transurethral resection of the prostate for symptomatic benign prostatic hyperplasia post 1994, with the potential for increased incidental diagnoses of early prostate cancer [21]. In addition, public funding of Prostate Specific Antigen (PSA) tests via Medicare became available from November 1993 [22]. Though its net public health value is extensively debated [23, 24], PSA testing has been commonly used in the community. Both changes increase the likelihood of earlier diagnosis and an increased incidence, potentially via diagnosis of cancers which would not otherwise be clinically significant [22, 25]. Since there were no substantive treatment advances during this period, changes in lead-time may be the predominant factor associated with the observed reduction in the probability of cancer death. The further marked decrease in the probability of prostate cancer death between 1998- 2002 and 2003–2007 is consistent with widespread introduction of adjuvant androgen deprivation for high risk early prostate cancer [26]; these changes mirror those seen in similar high-income countries [27].

Population based screening women for breast cancer in this jurisdiction began in 1989 [28]. Between 1985 and 1990, data emerged demonstrating the efficacy of tamoxifen as adjuvant therapy for early breast cancer [29–31]. The advent of screening is likely reflected in the > 10% increased incidence of female breast cancer observed in the second and to lesser extent third sub-periods, and more importantly in the substantial reduction in the probability of death from female breast cancer seen for patients diagnosed in this period. The reduction in the probability of female breast cancer death is most marked from 5 to 20 years post-diagnosis, suggesting that changes in diagnosis or management have prevented the development of metastatic, or fatal, breast cancer rather than merely delaying its onset. For patients diagnosed in subsequent periods, further reductions in cancer death may reflect the use and refinement of adjuvant chemotherapy for early breast cancer (reviewed in [32]).

For colorectal cancer the most marked reduction in the probability of cancer death parallels the introduction in 1990 of adjuvant chemotherapy for stage III colon cancer with 5-fluorouracil-based treatments [33], with subsequent uptake and long-term mortality reductions being delayed for the impact of ‘cure’ in disease that would have otherwise become metastatic. Treatment of stage II colon cancer with adjuvant chemotherapy became more widespread from 1995 onwards [34, 35]. Oxaliplatin-based adjuvant chemotherapy was introduced in 2004 [36] and this appears to be associated with further modest incremental reductions in the probability of death seen in periods after this time point. The growth of informal screening via colonoscopy and later introduction of formal faecal occult blood test screening is also likely to have played a role [37].

Lung cancer patients continued to present with advanced stage disease with poor prognosis [38]. The change in sex distribution observed in our study has previously been described [39–41] and is explained by the later peak prevalence for smoking for females relative to males in Australia [42]. A small reduction in the probability of death from lung cancer was observed after 2002, aligning with the introduction of adjuvant chemotherapy for resected non-small cell lung cancer [43], but may also be influenced by the increasing use of doublet platinum-based systemic therapy at that time [44]. The small reduction in probability of lung cancer death is seen both at 1 year after diagnosis, as well as in subsequent periods (5, 10, 20 years after diagnosis) suggest that both improved adjuvant and metastatic treatment are contributing.

Our finding of increased incidence and improved, though still high, probability of pancreatic cancer-death over time may be explained by concurrent increases in computed tomography use, which has increased incidental discovery of less-advanced disease. Even so, the high probability of cancer related death reflects late-stage presentation. The reduced probability of cancer death at 1 year following diagnosis may be partly explained by chemotherapy, which showed a small survival benefit in the late 1990s [45] and/or adjuvant chemotherapy following resection [46]. Similarly, in grade IV glioma the cumulative incidence of cancer related death did not decrease until 2003–2007; even then improvement was modest. This coincides with the introduction of combined chemoradiotherapy and adjuvant temozolomide (publicly funded in Australia in 2005 [47]), which showed improved survival [48].

WA provides an ideal location to study changes in the burden of cancer. Only 3% of individuals have any health records in another Australian state or territory [49] and there is good population data capture [10]. This means that though these data were not collected with this study aim in mind, the data used for this analysis have supported achieving the study aim. Newly implemented screening programs, new medical technology and new drugs are federally funded and made available concurrently for the whole population of Australia (even if roll-out is not uniform [50]), enhancing generalisability.

This study had some limitations. The allocation of sub-periods was uniform, rather than cancer-specific. While clinical practice changes have not occurred simultaneously for all cancer types, allocation of uniform sub-periods facilitated inter-cancer comparisons via the cumulative incidence function. No staging information was available in the data analysed. Cause of death coding is unlikely to be 100% accurate, especially for older persons [51]. For example, 593 of the 3,687 people dying in 2012 (0.3% of the study cohort), were censored as they had not yet been assigned a cause of death flag by the WA Cancer Registry at the time of data extraction. Coding is subject to quality assurance [52] and was cross-referenced with the ABS monthly until 2005 [53]; thus our broad interpretation at a population-level is unlikely to be affected. In terms of administrative data, it is the best source to use to achieve the study aim. Finally, factors such as over-diagnosis may account for some of the documented changes [54]. Detailed assessment of this is beyond the scope of this study, though should be considered when interpreting the study findings.

## Conclusions

Considering the justifiable expectation for confounder adjustment in observational epidemiological studies, standard methods for tracking population-level changes in cancer survival and death are simplistic. This study demonstrates how competing risks and sociodemographic covariates can be incorporated using readily available software. These estimates are conceptually more likely to reflect changes to cancer prevention, detection and management, compared with other survival measures where accounting for these factors is more difficult. While cancer has been focused on here, this technique has potential utility in survival analysis for other disease states with outcomes of interest subject to competing risks.

## Supplementary information

**Additional file 1.** Cancer types included for major cancer types combined cohort^#^

**Additional file 2.** Full characteristics of the cohort.

**Additional file 3.** Competing risk regression analysis of Western Australia cancer-specific mortality by time period of diagnosis for major cancer types combined and six selected cancer types with (A) and without (B) adjustment for comorbidities at diagnosis.

## Data Availability

The datasets generated and/or analysed during the current study are not publicly available due to agreements with the Western Australian Data Linkage Branch, Department of Health and the relevant data custodians.

## Abbreviations

SHR: Sub-distribution hazard ratio
WA: Western Australia
