# Supplementary material for: Incorporating competing risk theory into evaluations of changes in cancer survival: making the most of cause of death and routinely linked sociodemographic data
Source: BMC Public Health. 2020 Jun 26;20:1002. doi: 10.1186/s12889-020-09084-8 (PMC7318745; doi:10.1186/s12889-020-09084-8)
Supplement: Supplementary file 1 — Additional file 1. Cancer types included for major cancer types combined cohort# [file 12889_2020_9084_MOESM1_ESM.docx]

Additional File 1 Cancer types included for major cancer types combined cohort^#^

| Cancer | ICD/ICD-O-3 /Tumour Morphology Codes |
| --- | --- |
| Bladder & urinary tract | C65-C68 |
| Breast** | C50 |
| Cervix** | C53 |
| Colorectal | C18-C20, C218 |
| Glioma Grade I, II & III | Tissue morphology codes 9381, 9382, 9400, 9401, 9411, 9420, 9424, 9450, 9451 (unless with incorrect/irreconcilable grading, also note that these were not modelled separately in the individual cancer analysis). |
| Glioma Grade IV*** | Tumour morphology codes 9440, 9441, 9442 having tissue Grade code of 4 (anaplastic/undifferentiated) or coded as not determined. |
| Kidney | C64 |
| Laryngeal | C32 |
| Leukaemias (all) | 9800-9801, 9805, 9836-9837, 9823, 9820, 9826, 9827, 9831-9834, 9840, 9861, 9866-9867, 9870-9874, 9891,9895-9897, 9910,9920,9930-9931, 9863,9875-9876, 9860, 9940,9945-9946,9948 |
| Liver and intrahepatic bile ducts | C22 |
| Lung, bronchus, trachea | C33 |
| Lymphomas (all) | 9590, 9650-9667, 9670-9671, 9673, 9675, 9678-9680, 9684, 9687, 9689-9691, 9695, 9698-9699,9766, 9700-9702, 9705, 9708-9709, 9714, 9716, 9717-9719, 9727-9729, 9591, 9596-9599****, 9687 |
| Melanoma | C43; M-8720-8790 |
| Mesothelioma | M905; ICD10 C45 |
| Myeloma | 9731-9734 |
| Oesophageal | C15 |
| Ovarian** | C56 |
| Prostate* | C61 |
| Pancreatic | C25 |
| Stomach | C17 |
| Testicular* | C62 |
| Thyroid | C73 |
| Uterine (corpus)** | C54 |
| # Note the following cancer types were not included: in this study Lip, gum & mouth, tongue, parotid gland, major salivary gland (excluding parotid), pharynx, nasopharynx, small intestine, gallbladder and bile ducts, nasal cavity/sinuses/middle or inner ear, thymus, pleura, heart and mediastinum, bones, joints and articular cartilage, nervous system (peripheral/autonomic), peritoneum and retroperitoneum, connective and other soft tissues, vulva, vagina, uterine adnexa/other female genitalia, placenta, penis, male genitalia (other), adrenal gland, other chronic myeloproliferative disorders, polycythaemia ruba vera, myelofibrosis/sclerosis, chronic myeloproliferative disease not otherwise specified, malignant histiocytic/ dendritic cell neoplasm, other & unspecified immunoproliferative neoplasms, mast cell malignancies, refractory anaemias/cytopaenias, myelodysplastic syndromes, Kaposi’s sarcoma., non-malignant melanomas (excluding squamous cell carcinoma and basal cell carcinoma). | |
| *male only **female only  *** Tissue codes for the following 9440 (Glioblastoma, NOS or G. Multiforme, Spongioblastoma multiforme) 9441 (Giant cell glioblastoma, Monstrocellular sarcoma) 9442 (Glioblastoma /Sarcomatous, Glioblastoma w sarcomatous component, Gliofibroma (unc.), Gliosarcoma)  ****ICD-0-3 codes: 9597, 9598 and 9599 are Western Australian Cancer Registry codes for not otherwise specified non-Hodgkin Lymphoma which can be grouped as low, intermediate or high grade respectively but which would only be otherwise placed in the ICD-O classification as code 9591. | |
