# Supplementary material for: Incorporating competing risk theory into evaluations of changes in cancer survival: making the most of cause of death and routinely linked sociodemographic data
Source: BMC Public Health. 2020 Jun 26;20:1002. doi: 10.1186/s12889-020-09084-8 (PMC7318745; doi:10.1186/s12889-020-09084-8)
Supplement: Supplementary file 2 — Additional file 2. Full characteristics of the cohort. [file 12889_2020_9084_MOESM2_ESM.docx]

Additional File 2. Full characteristics of the cohort.

| **Characteristic** | | **Time period** | | | | | | | | | | | | **p-value*** |
| --- | --- | --- | --- | --- | --- | --- | --- | --- | --- | --- | --- | --- | --- | --- |
|  |  | **1983-1987** | | **1988-1992** | | **1993-1997** | | **1998-2002** | | **2003-2007** | | **2008-2011** | |  |
|  |  | **n** | **%** | **n** | **%** | **n** | **%** | **n** | **%** | **n** | **%** | **n** | **%** |  |
| **Major cancer types combined** | | | | | | | | | | | | | | |
| Follow up in years | Mean (SD) | 7.99 (9.86) | | 8.04 (8.59) | | 7.94 (6.98) | | 6.32 (4.93) | | 4.31 (2.84) | | 1.57 (1.18) | |  |
|  | Median (IQR) | 2.50 (13.78) | | 3.58 (17.47) | | 6.01 (14.47) | | 6.94 (9.92) | | 4.79 (5.38) | | 1.36 (2.01) | | <0.001 |
| Number of comorbidities at Dx | Mean (SD) |  |  |  |  |  |  | 1.87 (1.82) | | 1.70 (1.85) | | 1.51 (1.73) | |  |
|  | Median (IQR) |  |  |  |  |  |  | 1.00 (3.00) | | 1.00 (3.00) | | 1.00 (2.00) | | <0.001 |
| Sex | Female | 9,383 | 47.2 | 11,730 | 47.9 | 13,804 | 44.1 | 16,155 | 46.1 | 18,528 | 43.8 | 16,959 | 42.8 |  |
|  | Males | 10,499 | 52.8 | 12,747 | 52.1 | 17,481 | 55.9 | 18,880 | 53.9 | 23,777 | 56.2 | 22,698 | 57.2 | <0.001 |
| Age group at Dx | 0-10yrs | 113 | 0.6 | 117 | 0.5 | 128 | 0.4 | 145 | 0.4 | 129 | 0.3 | 131 | 0.3 |  |
|  | 11-17yrs | 77 | 0.4 | 72 | 0.3 | 123 | 0.4 | 108 | 0.3 | 118 | 0.3 | 90 | 0.2 |  |
|  | 18-24yrs | 246 | 1.2 | 239 | 1.0 | 272 | 0.9 | 277 | 0.8 | 313 | 0.7 | 262 | 0.7 |  |
|  | 25-44yrs | 2,132 | 10.7 | 2746 | 11.2 | 3,074 | 9.8 | 3341 | 9.5 | 3,441 | 8.1 | 3,006 | 7.6 |  |
|  | 45-64yrs | 6,635 | 33.4 | 7859 | 32.1 | 10,151 | 32.4 | 11,983 | 34.2 | 15,297 | 36.2 | 14,856 | 37.5 |  |
|  | 65-84yrs | 9,676 | 48.7 | 11 920 | 48.7 | 15,553 | 49.7 | 16,596 | 47.4 | 19,926 | 47.1 | 18,230 | 46.0 |  |
|  | 85+ yrs | 998 | 5.0 | 1 524 | 6.2 | 1,984 | 6.3 | 2,585 | 7.4 | 3,081 | 7.3 | 3,082 | 7.8 | <0.001 |
| Socio-economic status | Highest Disadvantaged | 3,523 | 17.7 | 4,351 | 17.8 | 5,362 | 17.1 | 6,390 | 18.2 | 7,858 | 18.6 | 4,464 | 11.3 |  |
|  | High Disadvantage | 5,729 | 28.8 | 7,552 | 30.9 | 7,579 | 24.2 | 6,557 | 18.7 | 7,191 | 17.0 | 8,947 | 22.6 |  |
|  | Moderate Disadvantage | 3,187 | 16.0 | 3,761 | 15.4 | 3,890 | 12.4 | 4,915 | 14.0 | 6,380 | 15.1 | 7,133 | 18.0 |  |
|  | Less Disadvantaged | 2,267 | 11.4 | 3,716 | 15.2 | 5,344 | 17.1 | 5,504 | 15.7 | 6,949 | 16.4 | 5,932 | 15.0 |  |
|  | Least Disadvantaged | 4,047 | 20.4 | 4,258 | 17.4 | 8,788 | 28.1 | 11,645 | 33.2 | 13,894 | 32.8 | 13,149 | 33.2 |  |
|  | Unknown | 1,129 | 5.7 | 839 | 3.4 | 322 | 1.0 | 24 | 0.1 | 33 | 0.1 | 32 | 0.1 | <0.001 |
| Accessibility to services | Very Remote | 451 | 2.3 | 521 | 2.1 | 666 | 2.1 | 727 | 2.1 | 948 | 2.2 | 970 | 2.4 |  |
|  | Remote | 284 | 1.4 | 353 | 1.4 | 470 | 1.5 | 564 | 1.6 | 665 | 1.6 | 611 | 1.5 |  |
|  | Accessible | 1,097 | 5.5 | 1,428 | 5.8 | 1,796 | 5.7 | 2,123 | 6.1 | 2,640 | 6.2 | 2,423 | 6.1 |  |
|  | Moderately Accessible | 1,138 | 5.7 | 1,354 | 5.5 | 1,621 | 5.2 | 1,853 | 5.3 | 2,036 | 4.8 | 2,019 | 5.1 |  |
|  | Highly Accessible | 16,755 | 84.3 | 20,688 | 84.5 | 26,690 | 85.3 | 29,750 | 84.9 | 35,998 | 85.1 | 33,618 | 84.8 |  |
|  | Unknown | 157 | 0.8 | 133 | 0.5 | 42 | 0.1 | 18 | 0.1 | 18 | 0.0 | 16 | 0.0 | <0.001 |
| **Total** |  | **19,882** | **10.3** | **24,477** | **12.7** | **31,285** | **16.2** | **35,035** | **18.2** | **42,305** | **22.0** | **39,657** | **20.6** |  |

| **Female breast cancer** | | | | | | | | | | | | | | |
| --- | --- | --- | --- | --- | --- | --- | --- | --- | --- | --- | --- | --- | --- | --- |
| Follow up in years | Mean (SD) | 11.88 (9.99) | | 12.88 (8.22) | | 11.70 (5.99) | | 9.24 (3.74) | | 5.81 (2.05) | | 1.88 (1.15) | |  |
|  | Median (IQR) | 8.81 (21.05) | | 14.01 (16.11) | | 14.43 (10.55) | | 10.72 (4.12) | | 5.93 (2.81) | | 1.77 (1.97) | | 0.001 |
| Number of comorbidities at Dx | Mean (SD) |  |  |  |  |  |  | 1.14 (1.43) | | 0.95 (1.37) | | 0.85 (1.28) | |  |
|  | Median (IQR) |  |  |  |  |  |  | 1.00 (2.00) | | 0.00 (1.00) | | 0.00 (1.00) | | 0.001 |
| Sex | Female | 2,599 | 100.0 | 3,444 | 100.0 | 4,379 | 100.0 | 5,238 | 100.0 | 5,820 | 100.0 | 5,527 | 100.0 | N/A |
| Age group at Dx | 25-44yrs | 463 | 17.8 | 634 | 18.4 | 710 | 16.2 | 737 | 14.1 | 776 | 13.3 | 694 | 12.6 |  |
|  | 45-64yrs | 1,071 | 41.2 | 1,497 | 43.5 | 2,025 | 46.2 | 2,689 | 51.3 | 3,110 | 53.4 | 2,821 | 51.0 |  |
|  | 65-84yrs | 939 | 36.1 | 1,129 | 32.8 | 1,441 | 32.9 | 1,567 | 29.9 | 1,662 | 28.6 | 1,720 | 31.1 |  |
|  | 85+ yrs | 122 | 4.7 | 179 | 5.2 | 197 | 4.5 | 237 | 4.5 | 270 | 4.6 | 287 | 5.2 | <0.001 |
| Socio-economic status | Highest Disadvantaged | 402 | 15.5 | 551 | 16.0 | 655 | 15.0 | 906 | 17.3 | 1,023 | 17.6 | 567 | 10.3 |  |
|  | High Disadvantage | 724 | 27.9 | 971 | 28.2 | 1004 | 22.9 | 959 | 18.3 | 927 | 15.9 | 1,145 | 20.7 |  |
|  | Moderate Disadvantage | 386 | 14.9 | 514 | 14.9 | 503 | 11.5 | 686 | 13.1 | 845 | 14.5 | 988 | 17.9 |  |
|  | Less Disadvantaged | 288 | 11.1 | 561 | 16.3 | 779 | 17.8 | 860 | 16.4 | 988 | 17.0 | 796 | 14.4 |  |
|  | Least Disadvantaged | 629 | 24.2 | 709 | 20.6 | 1,391 | 31.8 | 1,826 | 34.9 | 2,035 | 35.0 | 2,028 | 36.7 |  |
|  | Unknown | 170 | 6.5 | 138 | 4.0 | 47 | 1.1 | 1 | 0.0 | 2 | 0.0 | 3 | 0.1 | <0.001 |
| Accessibility to services | Very Remote | 42 | 1.6 | 52 | 1.5 | 89 | 2.0 | 123 | 2.3 | 127 | 2.2 | 127 | 2.3 |  |
|  | Remote | 37 | 1.4 | 46 | 1.3 | 65 | 1.5 | 73 | 1.4 | 68 | 1.2 | 67 | 1.2 |  |
|  | Accessible | 119 | 4.6 | 186 | 5.4 | 216 | 4.9 | 297 | 5.7 | 374 | 6.4 | 343 | 6.2 |  |
|  | Moderately Accessible | 148 | 5.7 | 155 | 4.5 | 213 | 4.9 | 248 | 4.7 | 243 | 4.2 | 242 | 4.4 |  |
|  | Highly Accessible | 2,224 | 85.6 | 2,982 | 86.6 | 3789 | 86.5 | 4,495 | 85.8 | 5,005 | 86.0 | 4,741 | 85.8 |  |
|  | Unknown | 29 | 1.1 | 23 | 0.7 | 7 | 0.2 | 2 | 0.0 | 3 | 0.1 | 7 | 0.1 | <0.001 |
| **Total** |  | **2,599** | **9.6** | **3,444** | **12.8** | **4,379** | **16.2** | **5,238** | **19.4** | **5,820** | **21.5** | **5,527** | **20.5** |  |
| **Colorectal cancer** | | | | | | | | | | | | | | |
| Follow up in years | Mean (SD) | 7.64 (9.36) | | 7.61 (8.12) | | 7.04 (6.61 | | 6.07 (4.79) | | 4.25 (2.74) | | 1.60 (1.16) | |  |
|  | Median (IQR) | 2.56 (12.68) | | 3.50 (13.83) | | 4.16 (13.48) | | 5.69 (9.45) | | 4.62 (4.99) | | 1.42 (1.98) | | 0.001 |
| Number of comorbidities at Dx | Mean (SD) |  |  |  |  |  |  | 2.24 (1.97) | | 2.16 (1.95) | | 2.11 (1.85) | |  |
|  | Median (IQR) |  |  |  |  |  |  | 2.00 (2.00) | | 2.00 (2.00) | | 2.00 (2.00) | | 0.49 |
| Sex | Female | 1,476 | 47.8 | 1,711 | 47.7 | 1,910 | 44.9 | 2,202 | 44.2 | 2,418 | 44.6 | 2,193 | 42.9 |  |
|  | Males | 1,612 | 52.2 | 1,875 | 52.3 | 2,344 | 55.1 | 2,776 | 55.8 | 3,005 | 55.4 | 2,917 | 57.1 | <0.001 |
| Age group at Dx | 25-44yrs | 130 | 4.2 | 171 | 4.8 | 182 | 4.3 | 213 | 4.3 | 207 | 3.8 | 223 | 4.4 |  |
|  | 45-64yrs | 1,068 | 34.6 | 1,172 | 32.7 | 1,373 | 32.3 | 1,528 | 30.7 | 1,751 | 32.3 | 1,680 | 32.9 |  |
|  | 65-84yrs | 1,692 | 54.8 | 1,969 | 54.9 | 2,322 | 54.6 | 2,719 | 54.6 | 2,910 | 53.7 | 2,670 | 52.3 |  |
|  | 85+ yrs | 193 | 6.3 | 271 | 7.6 | 369 | 8.7 | 510 | 10.2 | 534 | 9.8 | 522 | 10.2 | <0.001 |
| Socio-economic status | Highest Disadvantaged | 525 | 17.0 | 565 | 15.8 | 683 | 16.1 | 885 | 17.8 | 985 | 18.2 | 607 | 11.9 |  |
|  | High Disadvantage | 900 | 29.1 | 1,161 | 32.4 | 1,082 | 25.4 | 1,012 | 20.3 | 897 | 16.5 | 1,185 | 23.2 |  |
|  | Moderate Disadvantage | 513 | 16.6 | 539 | 15.0 | 513 | 12.1 | 708 | 14.2 | 886 | 16.3 | 933 | 18.3 |  |
|  | Less Disadvantaged | 321 | 10.4 | 588 | 16.4 | 719 | 16.9 | 721 | 14.5 | 891 | 16.4 | 740 | 14.5 |  |
|  | Least Disadvantaged | 657 | 21.3 | 606 | 16.9 | 1,221 | 28.7 | 1,651 | 33.2 | 1,757 | 32.4 | 1,638 | 32.1 |  |
|  | Unknown | 172 | 5.6 | 127 | 3.5 | 36 | 0.8 | 1 | 0.0 | 7 | 0.1 | 7 | 0.1 | <0.001 |
| Accessibility to services | Very Remote | 45 | 1.5 | 49 | 1.4 | 62 | 1.5 | 86 | 1.7 | 90 | 1.7 | 113 | 2.2 |  |
|  | Remote | 35 | 1.1 | 51 | 1.4 | 55 | 1.3 | 73 | 1.5 | 98 | 1.8 | 86 | 1.7 |  |
|  | Accessible | 165 | 5.3 | 213 | 5.9 | 228 | 5.4 | 319 | 6.4 | 334 | 6.2 | 324 | 6.3 |  |
|  | Moderately Accessible | 177 | 5.7 | 182 | 5.1 | 216 | 5.1 | 241 | 4.8 | 291 | 5.4 | 275 | 5.4 |  |
|  | Highly Accessible | 2,644 | 85.6 | 3,063 | 85.4 | 3,690 | 86.7 | 4,258 | 85.5 | 4,609 | 85.0 | 4,311 | 84.4 |  |
|  | Unknown | 22 | 0.7 | 28 | 0.8 | 3 | 0.1 | 1 | 0.0 | 1 | 0.0 | 1 | 0.0 | <0.001 |
| **Total** |  | **3,088** | **11.7** | **3,586** | **13.6** | **4,254** | **16.1** | **4,978** | **18.8** | **5,423** | **20.5** | **5,110** | **19.3** |  |
| **Lung cancer** | | | | | | | | | | | | | | |
| Follow up in years | Mean (SD) | 1.97 (4.75) | | 2.08 (4.36) | | 1.91 (3.70) | | 1.72 (2.94) | | 1.54 (2.08) | | 0.87 (0.91) | |  |
|  | Median (IQR) | 0.44 (1.09) | | 0.53 (1.27) | | 0.53 (1.29) | | 0.59 (1.34) | | 0.64 (1.65) | | 0.55 (1.10) | | <0.001 |
| Number of comorbidities at Dx | Mean (SD) |  |  |  |  |  |  | 2.16 (1.85) | | 2.01 (1.87) | | 1.87 (1.79) | |  |
|  | Median (IQR) |  |  |  |  |  |  | 2.00 (2.00) | | 1.00 (2.00) | | 1.00 (2.00) | | <0.001 |
| Sex | Females | 711 | 25.0 | 911 | 29.4 | 1,056 | 31.5 | 1,326 | 34.8 | 1,632 | 37.8 | 1,566 | 40.6 |  |
|  | Males | 2,130 | 75.0 | 2,191 | 70.6 | 2,301 | 68.5 | 2,488 | 65.2 | 2,685 | 62.2 | 2,291 | 59.4 | <0.001 |
| Age group at Dx | 25-44yrs | 59 | 2.1 | 70 | 2.3 | 65 | 1.9 | 79 | 2.1 | 63 | 1.5 | 65 | 1.7 |  |
|  | 45-64yrs | 1,096 | 38.6 | 1,036 | 33.4 | 951 | 28.3 | 1,027 | 26.9 | 1,146 | 26.5 | 1,073 | 27.8 |  |
|  | 65-84yrs | 1,596 | 56.2 | 1,857 | 59.9 | 2,137 | 63.7 | 2,431 | 63.7 | 2,753 | 63.8 | 2,292 | 59.4 |  |
|  | 85+ yrs | 89 | 3.1 | 136 | 4.4 | 198 | 5.9 | 272 | 7.1 | 348 | 8.1 | 418 | 10.8 | <0.001 |
| Socio-economic status | Highest Disadvantaged | 618 | 21.8 | 724 | 23.3 | 687 | 20.5 | 843 | 22.1 | 998 | 23.1 | 520 | 13.5 |  |
|  | High Disadvantage | 894 | 31.5 | 976 | 31.5 | 911 | 27.1 | 813 | 21.3 | 836 | 19.4 | 998 | 25.9 |  |
|  | Moderate Disadvantage | 454 | 16.0 | 511 | 16.5 | 415 | 12.4 | 514 | 13.5 | 664 | 15.4 | 737 | 19.1 |  |
|  | Less Disadvantaged | 305 | 10.7 | 406 | 13.1 | 594 | 17.7 | 595 | 15.6 | 710 | 16.4 | 572 | 14.8 |  |
|  | Least Disadvantaged | 440 | 15.5 | 381 | 12.3 | 719 | 21.4 | 1,044 | 27.4 | 1,107 | 25.6 | 1,028 | 26.7 |  |
|  | Unknown | 130 | 4.6 | 104 | 3.4 | 31 | 0.9 | 5 | 0.1 | 2 | 0.0 | 2 | 0.1 | <0.001 |
| Accessibility to services | Very Remote | 72 | 2.5 | 83 | 2.7 | 73 | 2.2 | 99 | 2.6 | 121 | 2.8 | 106 | 2.7 |  |
|  | Remote | 40 | 1.4 | 37 | 1.2 | 50 | 1.5 | 62 | 1.6 | 70 | 1.6 | 47 | 1.2 |  |
|  | Accessible | 176 | 6.2 | 165 | 5.3 | 196 | 5.8 | 238 | 6.2 | 281 | 6.5 | 245 | 6.4 |  |
|  | Moderately Accessible | 184 | 6.5 | 171 | 5.5 | 190 | 5.7 | 207 | 5.4 | 204 | 4.7 | 210 | 5.4 |  |
|  | Highly Accessible | 2,365 | 83.2 | 2,641 | 85.1 | 2,844 | 84.7 | 3,202 | 84.0 | 3,638 | 84.3 | 3,247 | 84.2 |  |
|  | Unknown | 4 | 0.1 | 5 | 0.2 | 4 | 0.1 | 6 | 0.2 | 3 | 0.1 | 2 | 0.1 | 0.34 |
| **Total** |  | **2,841** | **13.3** | **3102** | **14.6** | **3357** | **15.8** | **3814** | **17.9** | **4317** | **20.3** | **3857** | **18.1** |  |
| **Prostate cancer** | | | | | | | | | | | | | | |
| Follow up in years | Mean (SD) | 5.55 (6.11) | | 6.98 (6.50) | | 9.89 (6.04) | | 8.04 (4.07) | | 5.44(2.14) | | 1.90 (1.16) | |  |
|  | Median (IQR) | 3.45 (6.39) | | 4.68 (8.78) | | 10.30 (11.64) | | 9.43 (6.62) | | 5.54 (2.72) | | 1.87 (2.00) | | <0.001 |
| Number of comorbidities at Dx | Mean (SD) |  |  |  |  |  |  | 1.92 (1.72) | | 1.35 (1.68) | | 1.04 (1.42) | |  |
|  | Median (IQR) |  |  |  |  |  |  | 2.00 (3.00) | | 1.00 (2.00) | | 0.00 (2.00) | | <0.001 |
| Sex | Males | 1,765 | 100.0 | 2,589 | 100.0 | 5,417 | 100.0 | 4,684 | 100.0 | 7,697 | 100.0 | 7,919 | 100.0 |  |
| Age group at Dx | 25-44yrs | 5 | 0.3 | <5 | 0.2 | 6 | 0.1 | 14 | 0.3 | 36 | 0.5 | 49 | 0.6 |  |
|  | 45-64yrs | 248 | 14.1 | 402 | 15.5 | 1,408 | 26.0 | 1,526 | 32.6 | 2,875 | 37.4 | 3,258 | 41.1 |  |
|  | 65-84yrs | 1,364 | 77.3 | 1,911 | 73.8 | 3,680 | 67.9 | 2,822 | 60.2 | 4,378 | 56.9 | 4,258 | 53.8 |  |
|  | 85+ yrs | 147 | 8.3 | 272 | 10.5 | 323 | 6.0 | 322 | 6.9 | 408 | 5.3 | 352 | 4.4 | <0.001 |
| Socio-economic status | Highest Disadvantaged | 277 | 15.7 | 429 | 16.6 | 938 | 17.3 | 817 | 17.4 | 1,319 | 17.1 | 843 | 10.6 |  |
|  | High Disadvantage | 510 | 28.9 | 834 | 32.2 | 1,185 | 21.9 | 796 | 17.0 | 1,236 | 16.1 | 1,731 | 21.9 |  |
|  | Moderate Disadvantage | 295 | 16.7 | 395 | 15.3 | 668 | 12.3 | 696 | 14.9 | 1,156 | 15.0 | 1,348 | 17.0 |  |
|  | Less Disadvantaged | 219 | 12.4 | 393 | 15.2 | 946 | 17.5 | 713 | 15.2 | 1,246 | 16.2 | 1,156 | 14.6 |  |
|  | Least Disadvantaged | 375 | 21.2 | 456 | 17.6 | 1,621 | 29.9 | 1,659 | 35.4 | 2,735 | 35.5 | 2,836 | 35.8 |  |
|  | Unknown | 89 | 5.0 | 82 | 3.2 | 59 | 1.1 | 3 | 0.1 | 5 | 0.1 | 5 | 0.1 | <0.001 |
| Accessibility to services | Very Remote | 20 | 1.1 | 22 | 0.8 | 75 | 1.4 | 64 | 1.4 | 130 | 1.7 | 165 | 2.1 |  |
|  | Remote | 21 | 1.2 | 36 | 1.4 | 81 | 1.5 | 92 | 2.0 | 129 | 1.7 | 129 | 1.6 |  |
|  | Accessible | 109 | 6.2 | 177 | 6.8 | 316 | 5.8 | 314 | 6.7 | 509 | 6.6 | 472 | 6.0 |  |
|  | Moderately Accessible | 108 | 6.1 | 135 | 5.2 | 252 | 4.7 | 287 | 6.1 | 392 | 5.1 | 412 | 5.2 |  |
|  | Highly Accessible | 1,494 | 84.6 | 2,208 | 85.3 | 4,681 | 86.4 | 3,926 | 83.8 | 6,535 | 84.9 | 6,741 | 85.1 |  |
|  | Unknown | 13 | 0.7 | 11 | 0.4 | 12 | 0.2 | 1 | 0.0 | 2 | 0.0 | 0 | 0.0 | <0.001 |
| **Total** |  | **1,765** | **5.9** | **2,589** | **8.6** | **5,417** | **18.0** | **4,684** | **15.6** | **7,697** | **25.6** | **7,919** | **26.3** |  |
| **Pancreatic cancer** | | | | | | | | | | | | | | |
| Follow up in years | Mean (SD) | 0.82 (3.00) | | 0.80 (2.58) | | 1.05 (2.78) | | 1.00 (2.16) | | 0.90 (1.46) | | 0.73 (0.80) | |  |
|  | Median (IQR) | 0.18 (0.48) | | 0.23 (0.49) | | 0.31 (0.63) | | 0.35 (0.73) | | 0.38 (0.83) | | 0.44 (0.91) | | <0.001 |
| Number of comorbidities at Dx | Mean (SD) |  |  |  |  |  |  | 2.33 (1.94) | | 2.41 (2.12) | | 2.03 (1.89) | |  |
|  | Median (IQR) |  |  |  |  |  |  | 2.00 (2.00) | | 2.00 (3.00) | | 1.00 (2.00) | | <0.001 |
| Sex | Females | 218 | 46.0 | 261 | 49.3 | 331 | 47.8 | 413 | 51.1 | 504 | 49.0 | 448 | 46.7 |  |
|  | Males | 256 | 54.0 | 268 | 50.7 | 361 | 52.2 | 395 | 48.9 | 525 | 51.0 | 511 | 53.3 | 0.42 |
| Age group at Dx | 25-44yrs | 15 | 3.2 | 15 | 2.8 | 23 | 3.3 | 28 | 3.5 | 28 | 2.7 | 27 | 2.8 |  |
|  | 45-64yrs | 124 | 26.2 | 138 | 26.1 | 167 | 24.1 | 215 | 26.6 | 306 | 29.7 | 268 | 27.9 |  |
|  | 65-84yrs | 313 | 66.0 | 321 | 60.7 | 425 | 61.4 | 461 | 57.1 | 566 | 55.0 | 530 | 55.3 |  |
|  | 85+ yrs | 22 | 4.6 | 53 | 10.0 | 76 | 11.0 | 101 | 12.5 | 129 | 12.5 | 134 | 14.0 | 0.001 |
| Socio-economic status | Highest Disadvantaged | 89 | 18.8 | 100 | 18.9 | 131 | 18.9 | 152 | 18.8 | 197 | 19.1 | 103 | 10.7 |  |
|  | High Disadvantage | 140 | 29.5 | 159 | 30.1 | 183 | 26.4 | 173 | 21.4 | 187 | 18.2 | 237 | 24.7 |  |
|  | Moderate Disadvantage | 85 | 17.9 | 75 | 14.2 | 92 | 13.3 | 116 | 14.4 | 170 | 16.5 | 199 | 20.8 |  |
|  | Less Disadvantaged | 55 | 11.6 | 91 | 17.2 | 98 | 14.2 | 128 | 15.8 | 161 | 15.6 | 143 | 14.9 |  |
|  | Least Disadvantaged | 81 | 17.1 | 87 | 16.4 | 184 | 26.6 | 238 | 29.5 | 314 | 30.5 | 276 | 28.8 |  |
|  | Unknown | 24 | 5.1 | 17 | 3.2 | 4 | 0.6 | 1 | 0.1 | 0 | 0.0 | 1 | 0.1 | <0.001 |
| Accessibility to services | Very Remote | 5 | 1.1 | 11 | 2.1 | 20 | 2.9 | 9 | 1.1 | 18 | 1.7 | 18 | 1.9 |  |
|  | Remote | 8 | 1.7 | 5 | 0.9 | 12 | 1.7 | 9 | 1.1 | 18 | 1.7 | 15 | 1.6 |  |
|  | Accessible | 31 | 6.5 | 23 | 4.3 | 58 | 8.4 | 58 | 7.2 | 69 | 6.7 | 51 | 5.3 |  |
|  | Moderately Accessible | 39 | 8.2 | 33 | 6.2 | 43 | 6.2 | 46 | 5.7 | 39 | 3.8 | 43 | 4.5 |  |
|  | Highly Accessible | 390 | 82.3 | 456 | 86.2 | 559 | 80.8 | 685 | 84.8 | 884 | 85.9 | 832 | 86.8 |  |
|  | Unknown | 1 | 0.2 | 1 | 0.2 | 0 | 0.0 | 1 | 0.1 | 1 | 0.1 | 0 | 0.0 | 0.019 |
| **Total** |  | **474** | **10.6** | **529** | **11.8** | **692** | **15.4** | **808** | **18.0** | **1029** | **22.9** | **959** | **21.4** |  |
| **Grade IV glioma** | | | | | | | | | | | | | | |
| Follow up in years | Mean (SD) | 1.90 (5.34) | | 1.16 (3.19) | | 0.95 (2.41) | | 0.91 (1.49) | | 1.03 (1.36) | | 0.77 (0.67) | |  |
|  | Median (IQR) | 0.42 (0.89) | | 0.48 (0.81) | | 0.42 (0.79) | | 0.52 (1.49) | | 0.59 (1.08) | | 0.64 (0.84) | | 0.017 |
| Number of comorbidities at Dx | Mean (SD) |  |  |  |  |  |  | 2.39 (1.81) | | 2.66 (2.02) | | 2.75 (1.95) | |  |
|  | Median (IQR) |  |  |  |  |  |  | 2.00 (3.00) | | 2.00 (3.00) | | 3.00 (3.00) | | 0.14 |
| Sex | Females | 71 | 45.8 | 99 | 44.8 | 107 | 42.8 | 119 | 38.3 | 126 | 38.2 | 118 | 43.7 |  |
|  | Males | 84 | 54.2 | 122 | 55.2 | 143 | 57.2 | 192 | 61.7 | 204 | 61.8 | 152 | 56.3 | 0.34 |
| Age group at Dx | 25-44yrs | 20 | 12.9 | 20 | 9.0 | 20 | 8.0 | 28 | 9.0 | 16 | 4.8 | 12 | 4.4 |  |
|  | 45-64yrs | 71 | 45.8 | 109 | 49.3 | 104 | 41.6 | 131 | 42.1 | 146 | 44.2 | 118 | 43.7 |  |
|  | 65-84yrs | 61 | 39.4 | 81 | 36.7 | 110 | 44.0 | 137 | 44.1 | 154 | 46.7 | 130 | 48.1 |  |
|  | 85+ yrs | 0 | 0.0 | <5 | 1.4 | 10 | 4.0 | 11 | 3.5 | 10 | 3.0 | 7 | 2.6 | 0.018 |
| Socio-economic status | Highest Disadvantaged | 26 | 16.8 | 23 | 10.4 | 49 | 19.6 | 57 | 18.3 | 57 | 17.3 | 30 | 11.1 |  |
|  | High Disadvantage | 36 | 23.2 | 77 | 34.8 | 50 | 20.0 | 53 | 17.0 | 55 | 16.7 | 56 | 20.7 |  |
|  | Moderate Disadvantage | 23 | 14.8 | 40 | 18.1 | 31 | 12.4 | 45 | 14.5 | 48 | 14.5 | 47 | 17.4 |  |
|  | Less Disadvantaged | 27 | 17.4 | 30 | 13.6 | 43 | 17.2 | 53 | 17.0 | 50 | 15.2 | 52 | 19.3 |  |
|  | Least Disadvantaged | 31 | 20.0 | 45 | 20.4 | 73 | 29.2 | 103 | 33.1 | 120 | 36.4 | 85 | 31.5 |  |
|  | Unknown | 12 | 7.7 | 6 | 2.7 | 4 | 1.6 | 0 | 0.0 | 0 | 0.0 | 0 | 0.0 | <0.001 |
| Accessibility to services | Very Remote | 5 | 3.2 | 5 | 2.3 | <5 | 1.6 | <5 | 1.0 | 6 | 1.8 | 6 | 2.2 |  |
|  | Remote | <5 | 1.3 | 5 | 2.3 | 5 | 2.0 | 5 | 1.6 | 6 | 1.8 | 5 | 1.9 |  |
|  | Accessible | 7 | 4.5 | 9 | 4.1 | <5 | 1.6 | 22 | 7.1 | 22 | 6.7 | 10 | 3.7 |  |
|  | Moderately Accessible | 6 | 3.9 | 16 | 7.2 | 16 | 6.4 | 22 | 7.1 | 13 | 3.9 | 14 | 5.2 |  |
|  | Highly Accessible | 135 | 87.1 | 184 | 83.3 | 221 | 88.4 | 259 | 83.3 | 283 | 85.8 | 235 | 87.0 |  |
|  | Unknown | 0 | 0.0 | <5 | 0.9 | 0 | 0.0 | 0 | 0.0 | 0 | 0.0 | 0 | 0.0 | 0.1190 |
| **Total** |  | **155** | **10.1** | **221** | **14.4** | **250** | **16.3** | **311** | **20.2** | **330** | **21.5** | **270** | **17.6** |  |

DX = Diagnosis, SD = Standard deviation, IQR = Interquartile range, n = number of cases, % = percentage of total cancer type as specified.

*Two-sided p values calculated using Chi squared for categorical variables and Kruskal-Wallis test for continuous variables. This assessed changes between variables over the time period.

Cell counts with less than 5 cases with known socio-demographic characteristics are shown as <5 to preserve anonymity (as per the ethical requirements).

Note the number of individuals aged under 25 years of age is not shown for the individual cancer types due to very low numbers.
