# Supplementary material for: Incorporating competing risk theory into evaluations of changes in cancer survival: making the most of cause of death and routinely linked sociodemographic data
Source: BMC Public Health. 2020 Jun 26;20:1002. doi: 10.1186/s12889-020-09084-8 (PMC7318745; doi:10.1186/s12889-020-09084-8)
Supplement: Supplementary file 3 — Additional file 3. Competing risk regression analysis of Western Australia cancer-specific mortality by time period of diagnosis for major cancer types combined and six selected cancer types with (A) and without (B) adjustment for comorbidities at diagnosis. [file 12889_2020_9084_MOESM3_ESM.docx]

Additional File 3: Competing risk regression analysis of Western Australia cancer-specific mortality by time period of diagnosis for major cancer types combined and six selected cancer types with (A) and without (B) adjustment for comorbidities at diagnosis

| **Time period of diagnosis** | **Major cancer types combined^#^** | | | | **Female breast cancer** | | | | **Colorectal cancer** | | | | **Lung cancer** | | | |
| --- | --- | --- | --- | --- | --- | --- | --- | --- | --- | --- | --- | --- | --- | --- | --- | --- |
|  | **SHR** | **95% CI** | | **p*** | **SHR** | **95% CI** | | **p*** | **SHR** | **95% CI** | | **p*** | **SHR** | **95% CI** | | **p*** |
|  |  | **Lower** | **Upper** |  |  | **Lower** | **Upper** |  |  | **Lower** | **Upper** |  |  | **Lower** | **Upper** |  |
| **A. Models adjusted for sex, age, Indigenous status, socio-economic status, accessibility to services and number of comorbidities at diagnosis** | | | | | | | | | | | | | | | | |
| 1998-2002 | 1.00 | Reference | | | 1.00 | Reference | | | 1.00 | Reference | | | 1.00 | Reference | | |
| 2003-2007 | 0.84 | 0.82 | 0.86 | <0.001 | 0.81 | 0.73 | 0.90 | <0.001 | 0.87 | 0.81 | 0.93 | <0.001 | 0.89 | 0.84 | 0.94 | <0.001 |
| 2008-2011 | 0.86 | 0.83 | 0.89 | <0.001 | 1.06 | 0.91 | 1.23 | 0.48 | 0.88 | 0.81 | 0.96 | 0.003 | 0.95 | 0.89 | 1.00 | 0.06 |
| **B. Models adjusted for sex, age, Indigenous status, socio-economic status and accessibility to services at diagnosis** | | | | | | | | | | | | | | | | |
| 1998-2002 | 1.00 | Reference | | | 1.00 | Reference | | | 1.00 | Reference | | | 1.00 | Reference | | |
| 2003-2007 | 0.83 | 0.81 | 0.85 | <0.001 | 0.80 | 0.72 | 0.88 | <0.001 | 0.86 | 0.81 | 0.92 | <0.001 | 0.87 | 0.83 | 0.92 | <0.001 |
| 2008-2011 | 0.81 | 0.79 | 0.83 | <0.001 | 0.98 | 0.85 | 1.13 | 0.79 | 0.86 | 0.80 | 0.93 | <0.001 | 0.88 | 0.83 | 0.92 | <0.001 |
|  |  |  |  |  |  |  |  |  |  |  |  |  |  |  |  |  |
| **Time period of diagnosis** | **Prostate cancer** | | | | **Pancreatic cancer** | | | | **Grade IV glioma** | | | |  |  |  |  |
|  | **SHR** | **95% CI** | | **p*** | **SHR** | **95% CI** | | **p*** | **SHR** | **95% CI** |  | **p*** |  |  |  |  |
|  |  | **Lower** | **Upper** |  |  | **Lower** | **Upper** |  |  | **Lower** | **Upper** |  |  |  |  |  |
| **A. Models adjusted for sex, age, Indigenous status, socio-economic status, accessibility to services and number of comorbidities at diagnosis** | | | | | | | | | | | | |  |  |  |  |
| 1998-2002 | 1.00 | Reference | | | 1.00 | Reference | | | 1.00 | Reference | | |  |  |  |  |
| 2003-2007 | 0.79 | 0.71 | 0.87 | <0.001 | 0.92 | 0.82 | 1.03 | 0.15 | 0.77 | 0.65 | 0.92 | <0.004 |  |  |  |  |
| 2008-2011 | 0.69 | 0.59 | 0.80 | <0.001 | 0.85 | 0.76 | 0.95 | 0.004 | 0.69 | 0.57 | 0.84 | <0.001 |  |  |  |  |
| **B. Models adjusted for sex, age, Indigenous status, socio-economic status and accessibility to services at diagnosis** | | | | | | | | | | | | |  |  |  |  |
| 1998-2002 | 1.00 | Reference | | | 1.00 | Reference | | | 1.00 | Reference | | |  |  |  |  |
| 2003-2007 | 0.74 | 0.67 | 0.81 | <0.001 | 0.91 | 0.82 | 1.00 | 0.06 | 0.86 | 0.72 | 1.02 | 0.08 |  |  |  |  |
| 2008-2011 | 0.62 | 0.54 | 0.71 | <0.001 | 0.81 | 0.73 | 0.90 | <0.001 | 0.73 | 0.61 | 0.88 | 0.001 |  |  |  |  |

SHR = sub-distribution hazard ratio of cancer death (of specific cancer types for models with one cancer type).

*Fine and Gray competing risks regression model. All p-values are two-sided. ^#^Analysis for major cancer types combined was also adjusted for cancer type.
